# Correlation of Central Macular Thickness and the Best-Corrected Visual Acuity in Three Months After Cataract Surgery by Phacoemulsification and With Intraocular Lens Implantation

**DOI:** 10.7759/cureus.13856

**Published:** 2021-03-12

**Authors:** Ashjan Bamahfouz

**Affiliations:** 1 Ophthalmology, Umm Al-Qura University, Mecca, SAU

**Keywords:** cataract, optical coherence tomography, best corrected visual acuity, central macula thickness, cystoid macular edema

## Abstract

Purpose

To estimate the incidence of central macular edema (CME) following cataract surgery and to correlate the central macular thickness (CMT) to the best-corrected visual acuity (BCVA).

Methods

This cohort study in 2018-2019 included cataract grades I and II. They were operated by phacoemulsification and intraocular lens implantation. CMT was measured using spectral-domain optical coherence tomogram (OCT) before and for three months at one-month intervals after surgery. The change in BCVA and CMT were correlated at three months after surgery. Incidence of CME (more than two SD of pre-surgery CMT) was calculated.

Results

The mean CMT for 138 eyes operated for cataracts measured before and at one, two, and three months after uneventful surgery was 213 ± 24.9, 222.7 ± 25.5, 217.8 ± 34.8, and 215 ± 28.3 µ, respectively. The median BCVA at three follow-ups was 0.2 (interquartile range [IQR] 0.1; 0.2), 0.1 (IQR 0.0; 0.1), and 0.0 (0.0; 0.03), respectively. The incidence of CME at one and three months was 18% and 4.3%, respectively. The CMT and VA (LogMAR) one month after cataract surgery were significantly correlated (r = 0.4, Pearson P < 0.001). The visual improvement between one and two months post-surgery was not significantly correlated with CMT decline (r = 0.06, Pearson P = 0.5). The BCVA at one, two, and three months was 0.0 LogMAR in 28 (20.3%), 52 (37.7%), and 104 (75.4%) eyes, respectively. Linear regression model, age and diabetes are the risk factors at one month. At two and three months, no significant risk factors were found.

Conclusion

CME post-cataract surgery seems to be transient. CMT changes correlate with best-corrected vision changes and seem to be affected by age and presence of diabetes in the 1st month after surgery.

## Introduction

Cataract surgery using the phacoemulsification method and implantation of an intraocular lens within the capsular bag has been the most widely used surgical procedure by ophthalmologists during this decade [1]. Patients undergoing cataract surgery nowadays expect spectacle-free, functionally normal vision after surgery [2]. Therefore, any factor that could hamper the patient’s vision after successful modern cataract surgery must be identified through audit and addressed [3]. In eyes without posterior segment comorbidity, the hemodynamics of the posterior segment of the eye results in the accumulation of fluids in and around the macula after cataract surgery [4]. Use of topical anti-inflammatory eye drops for three months following cataract surgery has been found to be effective in reducing vision loss due to macular edema [5]. Detection of increased macular thickness as well as cystoid macular edema after cataract surgery in eyes with and without diabetes is efficient with the help of optical coherence tomography [6]. The three months following cataract surgery are very crucial for restoring the physiology of the eye in relation to surgical trauma caused during cataract surgery; therefore, cataract audits include indicators at three months after cataract surgery including the vision [7]. The risk of cystoid macular edema in patients without diabetic retinopathy but with diabetes was reported to be 80% higher than in those without diabetes [8]. The incidence of diabetes among adult Saudi patients who were scheduled for cataract surgery was as high as 37.5% in a province of southern Saudi Arabia [9]. Therefore, it will be interesting to study the rate of central macular edema (CME) among patients undergoing cataract surgery and its effect on vision in the short term after successfully completed surgery. 

To the best of our knowledge, the correlation of anatomic changes reflected by macular edema measured by optical coherence tomography (OCT) and visual acuity during three months after cataract surgery in otherwise healthy eyes of the Arab population has not to be carried out. 

The study site is a tertiary eye unit in western Saudi Arabia with a modern facility for eye care in an urban area. Annually, 550 to 600 cataract surgeries are carried out in the university hospital by qualified and experienced ophthalmic surgeons.

 We studied the incidence of CME measured by OCT at three monthly follow-ups after conventional cataract surgeries in eyes without ocular comorbidities during 2017-2019 and correlated with the best-corrected visual acuity (BCVA).

## Materials and methods

The institution ethical committee approved of this research project (KAMC 14-135). Patients undergoing conventional cataract surgery between 2017 and 2019 comprised the study population. Those with grade I (mild nuclear sclerosis, up to 10% cortical cataract obscuring pupillary area and up to 3% posterior capsular cataract in pupillary area ) or grade II cataracts (moderate nuclear sclerosis, up to 50% cortical cataract obscuring pupillary area and/or up to 30% posterior capsular cataract obscuring pupillary area) scheduled for phacoemulsification cataract extraction and intraocular lens implantation in capsular bag-and without other ocular comorbidities like uveitis, proliferative diabetic retinopathy, and glaucoma-were included in the study. Written consent was obtained for participation before and after surgery for the purpose of research. Those not agreeing to participate were excluded. The tenets of the Helsinki declaration were strictly observed during all steps of research.

We assumed that the incidence of cystoid macular edema after cataract surgery in eyes of populations with a high risk of diabetes would be 16% at one month and 8% at three months, [10] and that it would be significantly correlated with visual impairment. To achieve a 95% confidence interval and 90% power to the study with central macular thickness (CMT) measured before and at one month and three months after cataract surgery, we needed to study at least 127 eyes. To compensate for patients lost during follow-up, we elected to review 10% more cases. Thus, the final sample was 140 patients. We used the sample size calculation of the OpenEpi software for this purpose [11].

One cataract surgeon with experience in current methods of cataract surgery and with a logbook of more than 500 surgeries was involved in the preoperative assessment, surgeries, and postoperative follow-up for the present study.

Demographic information included the ages and genders of the cataract patients. Preoperative information included grade of cataract, presented visual acuity, and presence of diabetes. Visual acuity was assessed for each eye using the refraction workstation COS-6100.3100 (NIDEK Co LTD, San Jose, CA, USA). Visual acuity was noted using LogMAR notations. If vision could not be tested at a 6-meter distance, it was tried at 3-meter distance. The projection of light and perception of rays for eyes with vision more than 1.4 LogMAR were checked. CMT was measured using Spectralis optical coherence tomography (Heidelberg Engineering, Franklin, MA, USA) [12]. The mean surgery time was 30 minutes (starting to drape the eye to apply soft contact lens), and phaco time was less than 30 seconds with the machine set to deliver an average of 100 hyper-pulses per second [13].

Foldable intraocular lens (IOL) injector systems (Alcon, Oakland, NJ, USA) were used to insert foldable intraocular lenses of suitable power, and these were placed in the capsular bags. All patients were prescribed nonsteroidal anti-inflammatory (diclofenac sodium eye drops) three times daily for four weeks after cataract surgery and prednisolone 1% eye drops every four hourly for one week then tapering dose over four weeks. The detailed anterior segment assessment was carried out using a slit lamp bio-microscope (Topcon, Oakland, NJ, USA) before surgery and at each follow-up. CMT and BCVA for distance were also investigated and noted one, two, and three months after surgery. 

The difference in CMT across the three follow-ups was calculated using the preoperative OCT measurement as a reference. The change in the BCVA, however, was estimated based on the one-month BCVA as a reference since preoperative VA was uncorrected visual acuity (UCVA) for distance and most of the measurements were <0.1 LogMAR due to the presence of cataract. 

**Figure 1:**
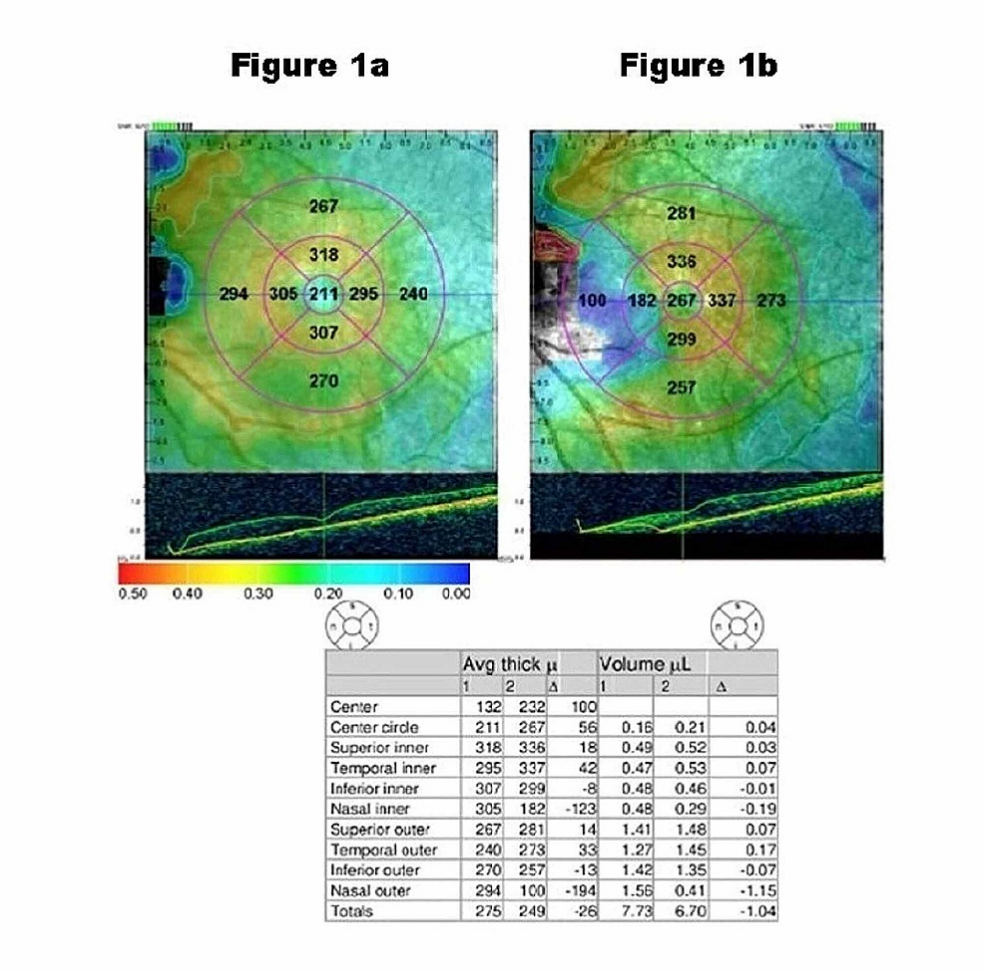
Optical coherence tomography of eye with cataract before (a) and one month after surgery (b).

CME was defined as two SDs above the preoperative mean CMT in OCT measurements. If the fluid was found in the retinal layers at the fovea, it was termed as cystoid macular edema (Figure 1b). The incidence of CME was calculated using total cataract surgeries as denominator, and the 95% confidence interval was calculated.

The data were collected on a pretested data collection form. The contents were transferred into the spreadsheet of the Statistical Package for Social Science (SPSS 25) (IBM, Armonk, NY, USA). The CMT at post-surgery, being a continuous variable, was reviewed for its distribution. Since it was normal, it was presented as mean and standard deviation. The comparison of outcomes in subgroups was performed using the parametric method of matched-pair analysis. The differences of mean, 95% confidence interval, and two-sided P-value were calculated. The BCVA was not normally distributed, so it was presented as median and interquartile range. The correlation of BCVA and CMT was performed using bi-variate method, and the r and Pearson P-value was calculated. Regression analysis was carried out using the linear regression model to study the effect of independent variables on the outcomes. A P-value of less than 0.05 was considered statistically significant.

## Results

We included all the 138 patients prospectively that were operated on the first eye to manage cataract. The mean age of patients was 65.5 ± 8.8 years. There were 65 males (47.1%), and 87 (63%) of right eyes were operated on. Diabetes was present in 48 (35%) patients. Their glycemic and other risk factors were controlled under the supervision of family physicians and endocrinologists beginning one month prior to the scheduled surgery. None of them had diabetic retinopathy or other posterior segment pathologies. 

During surgery, five patients had small anterior capsular tears or irregular capsulorhexis, but these issues were managed during surgery and IOLs were placed in the capsular bags without any vitreous disturbance. Only two out of five of these eyes had developed CME.

The incidence of CME at one and three months post-surgery was 18% and 4.3%, respectively. 

**Figure 2:**
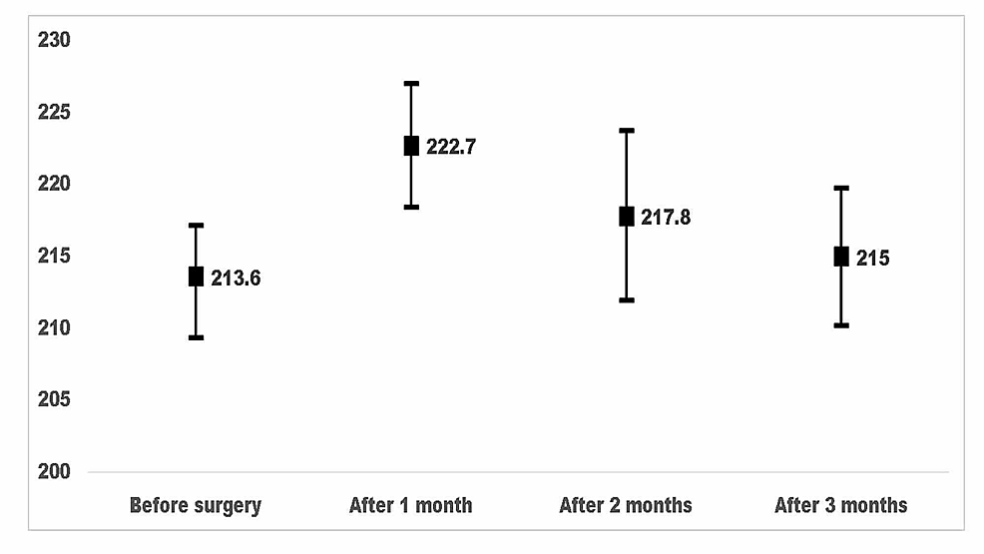
The central macular thickness before and at one-month interval after cataract surgery as measured with optical coherence tomography.

The CMT measurements before surgery and during the three follow-ups are given in Figure 2. The CME was significantly different at one month (P < 0.001) and two months after cataract surgery (P = 0.02) compared to before surgery, but it had resolved and reached preoperative status by three months after surgery (P = 0.35) [F = 2.75, P = 0.04].

The BCVA measurements at one, two, and three months after cataract surgery are compared in Table 1. The BCVAs at one, two, and three months were significantly different (P < 0.001). The BCVA was 20/20 (0.0 LogMAR) in 28 eyes (20.3%) at one month, 52 eyes (37.7%) at two months, and 104 eyes (75.4%) at three months after cataract surgery.

**Table 1:** Best-corrected visual acuity at different follow-up visits after cataract surgeries.

(N = 138)	Median	Interquartile range	Validation
(A) At 4 weeks after surgery	0.2	0.1; 0.2	r = 1
(B)At 8 weeks after surgery	0.1	0.0; 0.1	r = 0.67, Pearson P <0.001
(C) At 12 weeks after cataract surgery	0.0	0.0; 0.025	r = 0.72, Pearson P <0.001

We studied the effect of independent variables on CMT at one month and found that the increase in CMT at one month after cataract surgery was influenced by age (r = 0.18, P = 0.036), diabetes (P < 0.001), and preoperative CMT (r = -0.33, P < 0.001). But the increase in CMT at two months after cataract surgery was not influenced by age (r = 0.16, Pearson P = 0.06), gender (P = 0.13), eye involved (P = 0.23), diabetes (P = 0.06), or preoperative CMT (r = 0.18, P = 0.036). Similarly, the CMT at three months after cataract surgery was not influenced by age (r = -0.01, Pearson P = 0.06), gender (P = 0.06), eye involved (P = 0.6), diabetes (P = 0.8), or preoperative CMT (r = 0.12, Pearson P = 0.12).

We studied the effect of independent variables on CMT using linear regression model and found that at one month, the increase in CMT at one month after cataract surgery was influenced by age (P = 0.036), diabetes (P < 0.001), and preoperative central macular thickness (P < 0.001). But the increase in CMT at two months after cataract surgery was not influenced by age (P = 0.06), gender (P = 0.13), eye involved (P = 0.23), diabetes (P = 0.06), or preoperative CMT (r = 0.12, Pearson P = 0.12). Similarly, the CMT at three months after cataract surgery was not influenced by age (P = 0.27), gender (P = 0.06), eye involved (P = 0.6), diabetes (P = 0.8), or preoperative central macular thickness (P = 0.13).

We also compared the decline in CMT and improvement in vision at different follow-ups and found that the visual improvement between one and two months after cataract surgery was not significantly correlated to the decline in CMT (r = -0.13, Pearson P = 0.14). The visual improvement between the second and third month after cataract surgery was not significantly correlated with CMT (r = -0.1, Pearson P = 0.22).

## Discussion

Our study confirms that CMT and BCVA after conventional uneventful cataract surgery using the phacoemulsification technique and lens implantation seem to be interrelated. The incidence of CME is low. It is a transient phenomenon and seems to be influenced by the presence of diabetes. Since we used nonsteroidal anti-inflammatory medications postoperatively in all cases, we cannot confirm its role in the reduction of CMT.

The participants of the present study had cataracts of grades I and II. Generally, ophthalmologists of private sector and those of secondary eye care units in urban areas of industrialized countries manage early cataract cases to avoid complication in cataracts with hard nucleus. The resources and standard operating procedures used in our study to manage cataracts are also comparable to those available in other countries. Hence, the outcomes noted in the present study could be applied to eye patients undergoing modern cataract surgeries elsewhere. The incidence of central macular edema is dependent on the mode of its detection. If clinically detected, its rate is 0.1% to 2%. However, when it is detected using investigative tools like OCT or angiography, it also includes subclinical CME and its incidence ranges from 9% to 19% [14]. Both at one month and at three months after cataract surgery in the present study, the incidence of CME was within the range mentioned in the literature [11]. It was much lower than the clinically significant CME incidence reported in a large study of the European population [3]. Release of inflammatory mediators is postulated to be the reason for the development of CME after cataract surgery [15]. Such mediators are released less in non-diabetic compared to diabetic patients [16], in eyes with operative complications compared to uneventful cataract surgeries [17], and following small-incision cataract surgery compared to phacoemulsification cataract surgeries [18]. In view of all these risk factors related to CME (both clinical and sub-clinical), different professional bodies recommend topical non-steroidal anti-inflammatory drug (NSAID) application following cataract surgeries [12,19]. In the present study, all patients were treated with prophylactic NSAIDs as per institution protocol.

In the present study, we noted that CMT at one month was more thick than before surgery and this matched well with reduced BCVA. But as time passed after one month the CMT changes reduced but BCVA did not improve significantly. This implies that initially, edema (probably due to CME) might have been transient and related to a decrease in vision [20]. This component resolved by three months after cataract surgery, resulting in no correlation of BCVA with CMT. Keeping this in mind, one should counsel patients before surgery about anatomical and physiological changes taking place in eye after cataract surgery that can affect the vision. By using autorefractor that measures image reflection from central axial length for calculating the refractive error, one may make an error in the first few months when CMT is still changing. 

Presence of diabetes was a risk factor for increased CTM in cataract patients. This was also noted in the literature [21]. The patients with diabetic retinopathy (DR) in our study were excluded. All patients had HBA1c <6.5 at the time of surgery. Hence, risk factors of DR like poor glycemic control are less likely to influence the observed higher risk of CMT. The issue of subclinical CMT in diabetic patients in our study needs to be studied in more detail to reduce the risk of retinal complications after cataract surgery.

This study had a few limitations which are worth noting. The strict inclusion criteria and the exclusion of posterior segment pathologies and cataract grades III and IV resulted in a selective cataract population. The extrapolation of the study results to all cataract cases, especially those managed in rural locations with late presentation and in tertiary centers where cataracts with other ocular comorbidities are often managed, should be done with caution.

Cataract surgeries are widely performed ophthalmic surgeries worldwide. Patient expectations of remarkable visual recovery soon after cataract surgery are logical but not always possible. Issues that occur during the first three months post-surgery, such as transient CMT, need to be accounted for before making promises to patients who are expecting miracles to happen on the next day after cataract surgery. 

## Conclusions

In conclusion, CMT changes and visual recovery vary with time after cataract surgery and need close monitoring in each operated cataract cases; clinically through posterior segment evaluation using a stereoscopic slit lamp examination and, if needed, using noninvasive tools like OCT, in the 1st month after cataract surgery age and the presence of diabetes influence changes in CMT and BCVA.
